# ClusterONE Web: a tool for discovering and analyzing overlapping protein complexes

**DOI:** 10.1093/nar/gkaf368

**Published:** 2025-05-16

**Authors:** Marcelo Baez, Ruben Jimenez, Luca Cernuzzi, Alberto Paccanaro

**Affiliations:** Escola de Matemática Aplicada, Fundação Getúlio Vargas, Rio de Janeiro 22250-145, Brazil; Escola de Matemática Aplicada, Fundação Getúlio Vargas, Rio de Janeiro 22250-145, Brazil; Universidad Católica, “Nuestra Señora de la Asunción”, Asunción 001303, Paraguay; Centro de Ingeniería para la Investigación, Desarrollo e Innovación Tecnológica, Asunción 001303, Paraguay; Escola de Matemática Aplicada, Fundação Getúlio Vargas, Rio de Janeiro 22250-145, Brazil; Department of Computer Science, Centre for Systems and Synthetic Biology, Royal Holloway University of London, Egham TW20 0EX, UK

## Abstract

Protein–protein interactions (PPIs) are central to many cellular processes, and the assembly of proteins into complexes is essential for biological function. Clustering with overlapping neighborhood expansion (ClusterONE) has been successfully used to detect overlapping protein complexes in both weighted and unweighted PPI networks. Here, we present ClusterONE Web, a freely available, web-based tool that brings the functionality of ClusterONE into an accessible, user-friendly environment. The platform includes a database of preprocessed PPI datasets covering multiple organisms, reducing the need for manual data collection and preprocessing, while also allowing users to upload their own interaction data. Detected complexes are presented through an interactive interface, facilitating their exploration without requiring specialized software installation. The server also provides built-in Gene Ontology enrichment analysis to aid in the functional interpretation of identified complexes. ClusterONE Web is platform-independent and available at https://paccanarolab.org/clusteroneweb/.

## Graphical Abstract

**Graphical Abstract:**
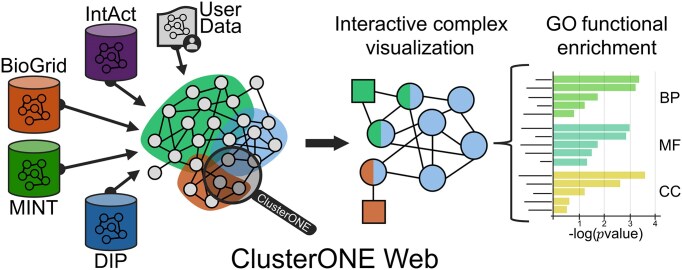

## Introduction

Protein complexes are fundamental units of cellular organization, driving essential biological processes such as signal transduction, gene regulation, and metabolic control[1, 2]. These complexes arise from physical interactions between proteins, forming functional assemblies that support various cellular mechanisms. Protein complexes can be identified from protein–protein interaction (PPI) networks, where they form densely interconnected clusters. However, standard clustering approaches that partition a graph into nonoverlapping clusters are not well suited for detecting protein complexes since proteins often participate in multiple clusters with distinct functions. Many clustering algorithms are also limited to unweighted networks and thus require some form of threshold-based filtering to handle weighted data. While individual edge weights can be difficult to assess, it has been shown that incorporating network weights significantly improves the identification of complexes [3].

Clustering with overlapping neighborhood expansion (ClusterONE) was introduced to address these challenges by detecting overlapping protein complexes in both weighted and unweighted PPI networks [3]. It grows clusters from “seed” vertices by greedily maximizing a cohesiveness measure that balances the density of internal edges against the connectivity to the rest of the network. This strategy identifies subgraphs that are densely interconnected and clearly separated from external regions of the network. Since its publication, ClusterONE has become widely used and recognized for its ability to capture the overlapping nature of protein complexes; however, the original implementation primarily relies on command-line operation, which may pose a constraint for many researchers who either lack the necessary computational expertise or prefer a more intuitive and graphical interface.

Here, we present ClusterONE Web, a freely available, web-based tool that brings the strengths of ClusterONE to a user-friendly environment. By integrating preloaded PPI networks from multiple public databases, ClusterONE Web enables researchers to explore protein complexes in diverse organisms without the overhead of installing specialized software or preprocessing different PPI datasets. The web server also performs an enrichment analysis of the identified complexes, offering functional insights based on Gene Ontology (GO) terms [4]. Additionally, users can upload their own PPI data for customized analysis, making ClusterONE Web a valuable resource for researchers studying the organization of protein complexes in diverse biological systems.

## Materials and methods

### ClusterONE

ClusterONE is an algorithm designed to detect overlapping protein complexes within PPI networks. The algorithm consists of three primary steps:

- *Greedy cluster growth*: Starting from an initial seed protein, ClusterONE employs a greedy strategy to expand the cluster. This expansion is guided by a cohesiveness measure that balances internal edge weights (interactions within the cluster) and boundary edge weights (interactions connecting the cluster to the rest of the network). Proteins are added or removed iteratively to maximize this cohesiveness, resulting in preliminary clusters.
- *Cluster merging*: Following the initial clustering, the algorithm assesses the overlap between clusters. Clusters exhibiting a high degree of overlap, as determined by a specified threshold, are merged to form unified clusters.
- *Postprocessing*: In the final step, the algorithm filters the clusters based on predefined criteria, such as minimum cluster size and density thresholds. Clusters that do not meet these criteria are discarded, refining the results to include only those clusters that are more likely to represent true protein complexes.

### Database of preloaded organisms

ClusterONE Web provides access to 99 preprocessed PPI datasets covering 58 organisms. These datasets are sourced from BioGrid [5], IntAct [6], MINT [7], and DIP [8], processed to conform to the ClusterONE format, and made readily available for analysis. In this initial release, only organisms with at least 50 reported interactions are included, and PPIs from viruses are excluded. This database also contains the necessary GO annotation files (GAF) for every organism to facilitate the enrichment analysis. To obtain the annotations, we first download the GAF for the entire UniProtKB from the EBI FTP server. The file was then preprocessed to generate individual GAFs for each organism included in ClusterONE Web. Details about the datasets used by ClusterONE Web, including the current version and detailed statistics regarding the number of proteins and interactions for each available PPI dataset, can be found in the official ClusterONE Web documentation at https://paccanarolab.org/clusteroneweb/doc/cl1web-docs.html#input-data.

### Gene Ontology enrichment analysis

ClusterONE Web attempts to attribute functions to each detected complex. This is done through over-representation analysis of GO terms associated with the proteins found in the complex. ClusterONE Web retrieves from the GAF file the list of GO terms that are associated with each protein in the complex—here only experimental evidence codes are used (i.e., EXP, IDA, IPI, IMP, IGI, IEP, TAS, and IC). The annotations are then up-propagated according to the true path rule, meaning that each annotation includes all its ancestral terms. To assess whether a GO term is statistically enriched in a complex, its frequency in the complex is compared against a reference background consisting of all annotated proteins of the same organism. Fisher’s exact test calculates *P*-values to determine the likelihood that the observed enrichment is due to chance. To account for multiple comparisons, *P*-values are adjusted using the Bonferroni correction. GO terms from each of the GO domains (Biological Process, Molecular Function, or Cellular Component) with significantly low *P*-values are considered over-represented, suggesting strong biological relevance for the complex.

### User-uploaded data

In addition to having access to the preloaded database of PPI networks, users can upload their own interaction networks in either TXT or CSV format, allowing the analysis of custom datasets. These files should follow a specific format, where each line consists of “id1 id2 weight,” with id1 and id2 representing the interacting proteins and weight indicating the confidence value between 0 and 1. The identifiers (id1 and id2) may be user-defined and do not need to correspond to known database identifiers such as UniProt IDs. If the weight is omitted, it is assumed to be 1, which applies to unweighted PPI networks. The columns of the input file may be separated by commas, spaces, or tabs; however, these separator characters should not be mixed within the same file. If interaction data are uploaded separately, users may also provide the corresponding GAF file. This step is optional but required for running the enrichment analysis on user-uploaded data.

### Web server backend and frontend design

The web tool is built on a modular backend architecture powered by Python FastAPI, chosen for its asynchronous capabilities and high performance in handling concurrent requests. We used Celery to parallelize the backend, and Redis as an in-memory cache to accelerate frequent database queries. All persistent data, such as preloaded PPI networks and ClusterONE results, are stored in a PostgreSQL relational database.

The frontend, built with React.js, communicates with the FastAPI backend via RESTful endpoints, ensuring seamless integration of visualization components (rendered using Cytoscape.js) with real-time updates from Celery tasks. Users receive immediate feedback on job statuses, with completed results streaming to the interface without page reloads.

This architecture achieves a balance between scalability (via Celery workers and Redis caching) and reliability (through PostgreSQL), making the tool suitable for both small-scale and large-scale PPI investigations.

## Results

### General overview

ClusterONE Web has been made accessible as a free and intuitive web server, hosted at https://paccanarolab.org/clusteroneweb/. The platform provides a user-friendly interface for selecting datasets from a collection of preloaded PPI databases spanning multiple organisms. This option drastically reduces the time needed to initiate analyses as it removes the need to manually collect and preprocess interaction data from different sources. Users can also upload their own PPI networks, with the option to provide a GAF file for the enrichment analysis.

After selecting or uploading a dataset, users can configure the parameters for running ClusterONE. Default settings are available for immediate use via the “Quick run ClusterONE” button, but parameters can be adjusted to fit specific requirements through the “Run ClusterONE” button. A link to the user manual is provided for detailed guidance on parameter selection and algorithm behavior.

**Figure 1.**
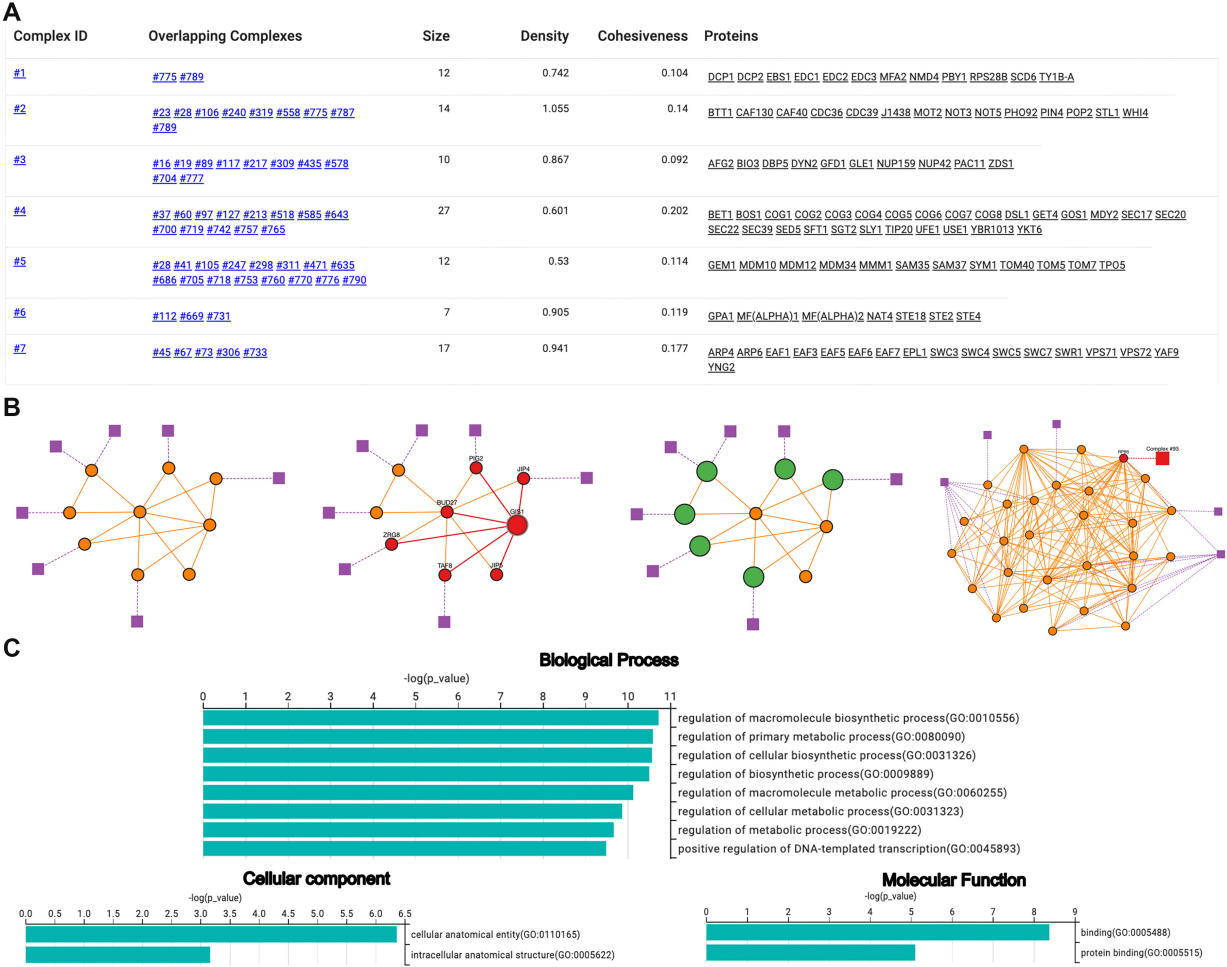
Results from running ClusterONE Web on the *Saccharomyces cerevisiae* (strain ATCC 204508/S288c) dataset using the default ClusterONE parameters. (**A**) Main results table listing the protein complexes detected by ClusterONE. For each complex, the table displays overlapping complexes, size, density, cohesiveness, and the associated proteins. (**B**) Examples of detected complexes visualized as interactive graphs. Orange circular nodes represent proteins, and purple rectangular nodes indicate overlapping complexes. From left to right: (i) default visualization of a protein complex; (ii) a selected protein is highlighted with increased node size, connectivity in red, and UniProt gene names displayed; (iii) overlapping proteins are highlighted in green; (iv) a selected overlapping complex is highlighted with increased node size and connectivity in red. (**C**) Enrichment analysis plots showing the top statistically significant terms for each domain: Biological Process, Cellular Component, and Molecular Function. The interactive graph examples correspond to Complex ID 93 (first three) and Complex ID 83 (last), and the enrichment analysis corresponds to Complex ID 93.

The results are displayed on the main results page as a structured table listing the identified protein complexes, as shown in Fig. 1A. Users can examine the composition and properties of each complex, with options to filter, search, and sort the table. For instance, complexes can be sorted by size or filtered within a specific size range. Users can also search for complexes containing a particular protein or group of proteins, allowing for an efficient exploration of the results.

If a complex overlaps with other complexes, the corresponding complex IDs are listed in the “Overlapping Complexes” column; otherwise, this column is left empty. This table also serves as an entry point for visualizing complexes interactively by simply clicking on a complex ID. The selected cluster is displayed as an interactive graph, letting users explore protein interactions with full control over the network elements. As shown in the examples in Fig. 1B, circle nodes represent proteins, while edges correspond to interactions. Overlapping complexes are indicated by square nodes in a distinct color: a connection between a protein and a square node indicates that the protein is shared with that complex. To navigate between overlapping complexes, a double-click on an overlapping complex node loads the corresponding complex, without the need to return to the initial results table. The platform also allows users to highlight nodes to inspect connectivity or analyze overlapping proteins. When hovering over or clicking on a protein, the official UniProt gene name is displayed. If no gene name is available, the open reading frame (ORF) name is provided instead. Double-clicking on a protein node opens its UniProt entry, providing direct access to additional annotations.

In parallel, the enrichment analysis becomes readily available for all complexes with at least one over-represented GO term. The results are accessible through the “Enrichment Analysis” button and are visualized in bar plots covering the three GO domains: Biological Process, Molecular Function, and Cellular component, as shown in Fig. 1C. The platform also includes a “Search by GO term” option, allowing users to find complexes based on their annotations, providing a way to explore the results from a functional perspective.

We provide the option for downloading the overall ClusterONE results as a CSV table, and for each complex, users can download publication-quality images (600 dpi) and a PDF containing the enrichment analysis.

### Illustrative example

**Figure 2.**
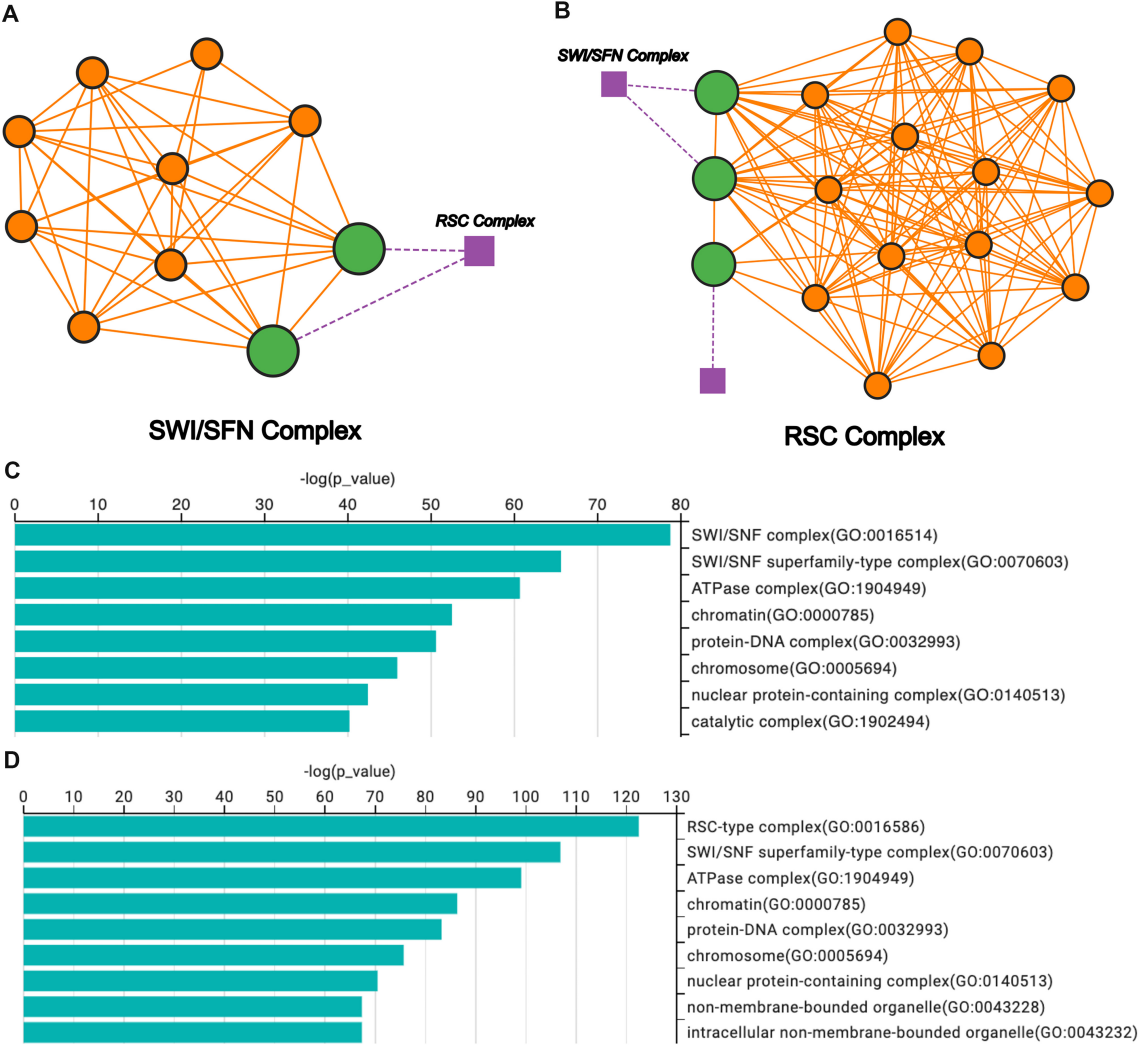
(**A**) Interactive graph of the SWI/SNF complex, as displayed by ClusterONE Web. (**B**) Interactive graph of the RSC complex, visualized in the same interface. In both subfigures, overlapping proteins are shown in green. (**C**) Cellular Component enrichment plot for the SWI/SNF complex. (**D**) Cellular Component enrichment plot for the RSC complex, illustrating the principal overrepresented GO categories, which align with the known subcellular localization of these complexes. The SWI/SNF complex corresponds to Complex ID 19 and the RSC complex to Complex ID 21.

To illustrate the capabilities of ClusterONE Web, we ran ClusterONE using the default parameters on the *S. cerevisiae* dataset from Collins *et al.* [9] and analyzed a well-studied chromatin remodeling module. Figure 2A and 2B shows two complexes, as identified by ClusterONE, corresponding to the SWI/SNF and RSC complexes. ClusterONE correctly identified the two overlapping proteins between these two complexes, and they can be easily visualized in the platform highlighted in green.

These complexes are well-characterized ATP-dependent chromatin remodelers involved in nucleosome repositioning and transcriptional regulation [10, 11]. Figure 2C and 2D reveals significant cellular component terms associated with the SWI/SNF and RSC complexes, respectively, as well as ATPase-related terms, consistent with their known functions. To replicate these results, users can click on the “Run Quick Example” button on the main menu of ClusterONE Web.

## Discussion

We present ClusterONE Web, a web-based platform for identifying and analyzing protein complexes. It integrates the ClusterONE algorithm with interactive visualization and functional enrichment analysis, allowing users to explore protein complexes without the need for local installation. We also provide a database of preloaded datasets from multiple sources, offering a selection of organisms for users to choose from and the option to upload custom PPI networks and GO annotations.

Different tools have been developed for protein complex detection and visualization, each offering varying levels of functionality, customization, and accessibility. Cytoscape-based plugins benefit from integration within the Cytoscape ecosystem but require local installation [12, 13]. Web-based platforms provide immediate access without a specialized setup but are often designed for specific organisms, datasets, or types of analysis [14–16]. In contrast, ClusterONE Web integrates the ClusterONE algorithm into a one-stop, browser-accessible platform that supports PPI network-based clustering from a broad range of organisms, as well as specific user-provided datasets, interactive graph exploration, and functional enrichment analysis.

ClusterONE Web is platform-independent and designed for ease of use, making it accessible to researchers across different computational backgrounds. While it already provides access to preprocessed PPI datasets from multiple organisms, its modular architecture and API-based design facilitate the integration of additional interaction networks and annotation sources, further expanding its analytical capabilities. ClusterONE Web was primarily designed for the detection of intra-species protein complexes from curated PPI networks. In the future, as more inter-species interaction datasets become available, our tool could potentially be used to explore host–pathogen interactions and their role in disease. As a potential direction for future work, a usability study could help identify areas for improvement and enhance the user experience. This platform offers a practical and valuable resource for exploring protein complexes and advancing biological research across diverse datasets.

## Data Availability

The source code for the ClusterONE Web web server can be found at https://doi.org/10.6084/m9.figshare.28466486. The data underlying this article are publicly available from BioGRID, IntAct, MINT, and DIP. The datasets were obtained from these sources and integrated into ClusterONE Web for analysis. The platform is free and open to all users and there is no login requirement. The tool is available at https://paccanarolab.org/clusteroneweb/.
